# Practices and barriers in screening for cognitive impairment in heart failure: a clinician survey

**DOI:** 10.1093/eschf/xvag212

**Published:** 2026-08-06

**Authors:** Sotiria Liori, Chris J Kapelios, Kalliopi Keramida, Stamatis Adamopoulos, Apostolos Karavidas, Sotirios Xydonas, Antonis Sideris, Christina Chrysohoou, John Parissis, Taskforce of the Hellenic Heart Failure Clinics Network, Ioannis Alexanian, Athanasios Anadiotis, Xanthi Apostolidou, Soultana Bakaimi, Constantinos Bakogiannis, Despoina Balta, Dora Bambali, Michael Bonios, Alexandros Briasoulis, Georgios Chalikias, Eftychia Chamodraka, Christos Chasikidis, Christos Chatzieleftheriou, Argirios Dalianis, Georgios Diakakis, Leonidas Diamantopoulos, Stella Dourtsiou, Metaxia Driva, Dimitrios Farmakis, Gerasimos Filippatos, Katerina Fountoulaki, Nikolaos Fragakis, Gerasimos Gavrielatos, Maria Georgopoulou, Grigoris Giamouzis, George Giannakoulas, George Giannoglou, Nikolaos Gouliaros, Angeliki Gkouziouta, Nikolaos Kalantzis, Vasileios Kamperidis, Nikolaos Kampouridis, Iraklis Kapitsinis, Theodoros Karamitsos, Eleni Karapatsoudi, Athanasios Kartalis, Anastasia Katinioti, Konstantinos Katsilis, Anastasia Kitsiou, Anna Kotsia, Maria Kouli, Nikos Kouris, Stauroula Kosmopoulou, Emmanouil Lamprogiannakis, Stylianos Lampropoulos, Evangelos Lazaris, Ioannis Leontsinis, Athanasios Manginas, Polyxeni Mantzouratou, Spyridon Maragkoudakis, Maria Marketou, Georgios Nikitas, Petros Nikolopoulos, Paraskevi Ntoliou, Christodoulos Papadopoulos, Dimitrios Papadopoulos, Konstantinos Papadopoulos, Georgios Papanikolaou, Minas Pappas, Stefanos Papastefanou, Vasilios Sarakis, Evangelos Sdogkos, Maria Sifaki, Vasiliki Sklirou, Georgios Spyromitros, Alexia Stavrati, Ioannis Stratakis, Maria Stratinaki, Efstratios Theofilogiannakos, Maria Thodi, Konstantina Toli, Konstantinos Tsatiris, Georgios Tsinopoulos, Konstantinos Tsiptsis, Konstantinos Tsitlakidis, Dimitrios Tziakas, Elias Tsougos, Vasileios Vasilakopoulos, Vassilios Vassilikos, Athanasios Vasilopoulos, Niki Vlassopoulou, Ioannis Vogiatzis, Petros Voutas, Alexandros Xanthopoulos, Ermioni Zachariadi, Antonios Ziakas

**Affiliations:** Heart Failure Unit, Department of Cardiology, Athens University Hospital Attikon, National and Kapodistrian University of Athens School of Medicine, Rimini 1, Athens 12462, Greece; Heart Failure Unit, Department of Cardiology, Athens University Hospital Attikon, National and Kapodistrian University of Athens School of Medicine, Rimini 1, Athens 12462, Greece; Department of Cardiology, General Anti-Cancer Oncological Hospital Agios Savvas, Athens, Greece; Heart Failure and Transplant Units, Onassis Hospital, Athens, Greece; Department of Cardiology, ‘G. Gennimatas’ General Hospital, Athens, Greece; Department of Cardiology, Evaggelismos Hospital, Athens, Greece; Department of Cardiology, Evaggelismos Hospital, Athens, Greece; First Department of Cardiology, Hippokration General Hospital of Athens, National and Kapodistrian University of Athens, Athens, Greece; Heart Failure Unit, Department of Cardiology, Athens University Hospital Attikon, National and Kapodistrian University of Athens School of Medicine, Rimini 1, Athens 12462, Greece

**Keywords:** heart failure, cognitive impairment, cognitive screening, clinician survey

## Abstract

**Background:**

Cognitive impairment (CI) affects a significant percentage of heart failure (HF) patients, reducing adherence and worsening outcomes. Routine cognitive assessment remains limited.

**Objectives:**

To evaluate cognitive assessment practices, identify barriers, and examine associations between knowledge, attitudes, and clinical behaviours among clinicians managing HF patients.

**Methods:**

An anonymous cross-sectional survey was distributed to 120 cardiologists across 68 clinics in the Hellenic Heart Failure Clinics Network (May–June 2025). The 13-question survey examined knowledge on cognitive decline, personal opinions on screening, assessment methods, and implementation obstacles. Associations were examined using Pearson’s chi-square.

**Results:**

Fifty-one cardiologists completed the survey, corresponding to a response rate of 42.5%. Among respondents 80% reported awareness of the high prevalence of cognitive decline, 14% systematically screened for CI presence. Main barriers reported were lack of time (76%) and inadequate familiarity with assessment tools (50%). Screening frequency was not associated with HF patient volume (χ^2^ = 4.75, *P* = .58). Significant correlations were demonstrated between (i) knowledge on CI and frequency of screening (*V* = 0.37, *P* = .04); (ii) frequency of screening and support of establishing screening as routine practice (*V* = 0.50, *P* < .001); and (iii) frequency of screening and referral of patients to specialists (*V* = 0.39, *P* = .007). All respondents expressed interest in further education.

**Conclusion:**

There is a gap between awareness of and testing for CI in HF patients, with the latter not being affected by clinicians’ patient load. Since there is universal interest in learning, integrating screening tools into daily routines and creating clear referral pathways may help in addressing this evidence-practice gap.

## Introduction

Advances in heart failure (HF) therapy have increased survival, elevating the focus on managing complex, non-cardiac comorbidities, among which cognitive impairment (CI) possesses a central spot. CI is a frequent and recognized comorbidity in this population, which is associated with a higher risk of death and HF readmissions,^1–6^ likely because its presence compromises the high level of self-care and adherence required for optimal HF outcomes.^7,8^ Impaired memory and attention specifically affect compliance with complex medication regimens, dietary restrictions, and early symptom recognition, leading to higher rates adverse events.^3,9^ CI in HF is multifactorial, involving cerebral hypoperfusion, thrombo-embolic episodes, inflammation, and neurohumoral mechanisms.^7,10–13^

Despite the recognized prevalence and adverse impact of CI, the clinical approach to diagnosis remains challenging and often unstructured,^14–16^ as there is a lack of standardized screening methods, contributing to a substantial implementation gap. CI is often under-recognized, particularly in the acute setting, as the absence of systematic guidelines hinders universally integrated screening.

This survey examined clinicians’ knowledge on CI, current screening practices, and main obstacles in CI screening.

## Methods

**Figure 1:**
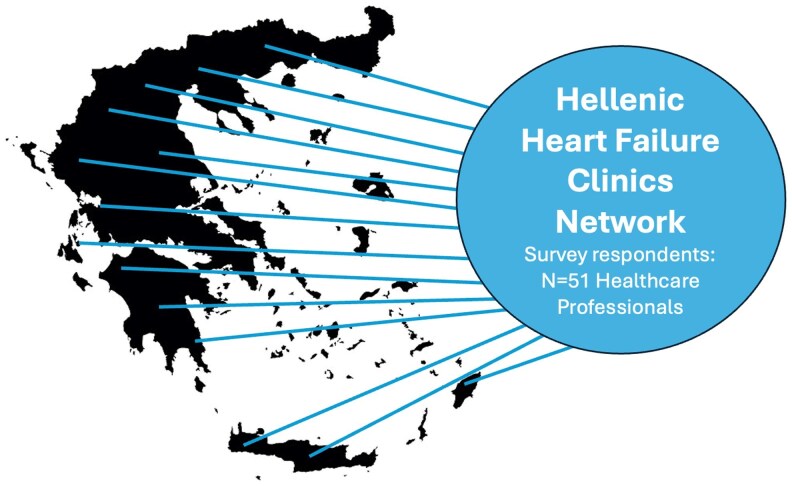
Nationwide distribution of heart failure clinics in Greece. Blue lines illustrate the broader geographic reach of the Hellenic Heart Failure Clinics Network. Survey respondents were anonymous clinicians recruited from this network (*N* = 51), representing diverse geographic regions and clinical settings across Greece

This was an anonymous, cross-sectional survey conducted between May and June 2025 to evaluate cognitive assessment practices, barriers, and attitudes among clinicians managing HF patients. The survey was distributed to 120 cardiologists across the 68 clinics of the Hellenic Heart Failure Clinic Network. The geographically representation of the HF clinics is shown in *Figure 1*.

We used a 13-item close-ended questionnaire, structured in Greek, covering six domains: professional characteristics, knowledge and awareness, assessment practices, methods and tools, barriers, and system integration. Data were collected electronically via a secure platform.

Descriptive statistics are shown as frequencies and/or percentages. Correlations between categorical variables were examined using Pearson’s chi-square tests (two-sided, α = .05). For significant associations, the χ^2^ statistic, df, *P*-value, and n were reported. Effect sizes for chi-square tests were calculated using Cramér’s *V*, which quantifies the strength of association between categorical variables and ranges from 0 (no association) to 1 (perfect association).

## Results

**Figure 2:**
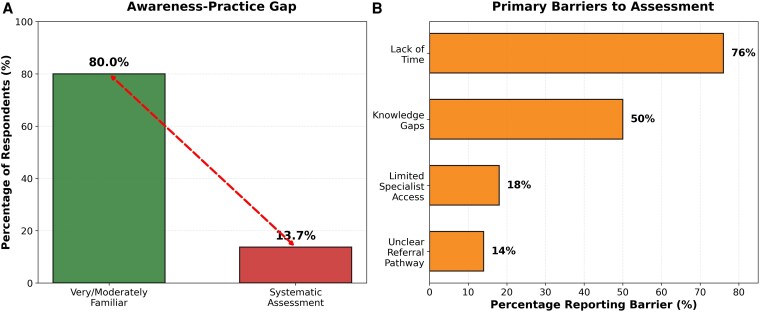
(*A*) Despite 80% familiarity with cognitive decline in HF, only 13.7% of healthcare professionals assess systematically. The red arrow highlights the substantial gap between awareness and practice (*B*) Primary barriers include lack of time (76%) and knowledge gaps (50%). *N* = 51 clinicians

A total of 51/120 (42.5%) cardiologists completed the survey. The majority (78%) reported daily HF encounters. Most participants (80%) reported being moderately to very familiar with CI, and 80% believed CI was quite to very common (*Figure 2*). Only 13.7% reported systematic assessment of CI, while 56.9% assessed CI occasionally, when its presence was suspected. Regarding methods and tools, informal clinical assessment (43.1%) and specialist referral (45.1%) were the most common approaches, while the use of formal tools [Mini-Mental State Examination (MMSE), Montreal Cognitive Assessment (MoCA), or other] totalled 54.9%. The primary reported barriers to CI assessment were lack of time (76%) and lack of knowledge about tools (50%), whereas only 2% considered assessment unnecessary. Most respondents (92.2%) recognized the impact of CI on reduced adherence, and 56.9% supported systematic integration of screening into routine care. Educational interest in CI was universal (100%), yet only 8% reported referring patients for further evaluation on a regular basis.

The most important correlations noted were:

- Clinicians who frequently assess patients and recognize the prevalence of the problem are more likely to refer patients for help (*V* = 0.39, *P* = .007).
- Clinicians who already assess patients support making screening a routine part of care (*V* = 0.50, *P* < .001).
- Physician patient volume—stratified by daily, weekly, or monthly HF encounters—was not associated with the frequency of screening for CI in this patient population (χ^2^ = 4.75, *P* = .58).

On the other hand, rates of routine screening support (*P* = .77) or referral (*P* = .13) did not differ significantly between cardiologists reporting familiarity with CI vs not.

## Discussion

**Figure 3:**
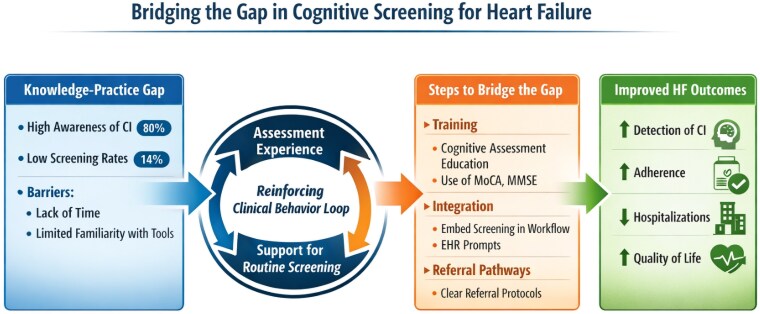
Illustration of the gap between awareness and implementation of cognitive impairment (CI) screening in heart failure (HF) care. Although physicians’ awareness of CI is high, systematic screening remains limited due to time constraints and lack of familiarity with assessment tools. Experience with cognitive assessment reinforces both referral practices and support for routine screening. Integration of training, workflow redesign, and structured referral pathways may facilitate implementation, ultimately improving adherence, clinical outcomes, and quality of life in HF patients

This study provides quantitative data on cognitive assessment practices in HF care. By capturing knowledge, attitudes, practices, and barriers, we identified a substantial knowledge-practice gap (*Figure 3*).

Namely, while 80% of respondents recognized the importance of CI, only 14% reported performing systematic assessments. This rate aligns with international reports of underrecognition and stands in contrast to the known prevalence of CI, which ranges between 25% and 75% in the HF population.^1,2^ Given the prognostic significance of CI,^3–5,7–9^ bridging this gap is critical.

On a clinical level, our data reveal a reinforcing cycle: clinicians who systematically assess CI are more likely to refer patients for specialized care and support screening integration. This suggests that experience validates practice. Consequently, clinical teams might implement a phased approach, initially targeting high-risk patient groups—such as those with polypharmacy, frequent hospitalizations, or evident self-care deficits—before attempting universal screening, aiming at building confidence and expanding adoption.

Furthermore, we showed that awareness on CI alone did not predict routine screening support, suggesting that interventions relying solely on education are insufficient without practical implementation measures. On the other hand, our findings also bring up an interesting contradiction: though 76% of respondents cited lack of time-consistent with the complex, time-limited nature of routine HF encounters—as one of the main barriers to CI assessment, physician patient load was not significantly correlated with frequency of testing, highlighting that system changes uncoupled with educational activities are unlikely to effect the desired results.

Collectively, our findings suggest that in order to address these structural barriers, healthcare systems must prioritize workflow integration combined with educational mandates. The former involves embedding brief screening tools directly into protocols with designated time allocations and utilizing electronic health records (EHR) prompts to standardize the process. The latter involves targeted training programmes to ensure clinicians recognize the high prevalence of CI and are competent in utilizing assessment tools. Additionally, shifting the burden of initial screening to trained non-physician staff could alleviate time pressures, addressing the bottlenecks reported by clinicians.

Future research should test implementation strategies to improve cognitive screening in HF. Randomized studies could compare education alone versus education plus workflow support, different tools (Mini-Cog, MoCA, MMSE), nurse- vs physician-led models, and EHR prompts versus usual care, focusing on screening rates, feasibility, detection, and downstream referrals and outcomes. Ultimately, outcome and economic evaluations—such as trials with formal cost-effectiveness analyses—are essential to determine whether screening improves adherence, self-care, hospitalizations, mortality, and quality of life in a way that justifies the required resources.

Several limitations warrant consideration. Convenience sampling from professional networks may introduce selection bias towards more engaged clinicians, potentially overestimating assessment rates compared to the broader population. The modest sample size and specific geographic context limit generalizability. Self-reported practices may not reflect actual behaviour due to social desirability bias (overreporting assessment frequency) or recall bias (inaccurate memory of practices). Chart review or direct observation would provide more objective measurement but was beyond this study’s scope. Importantly, the study was conducted in a single country and language, limiting cross-cultural applicability, though cognitive assessment barriers are likely shared across healthcare systems with similar resource constraints. This survey did not assess patient outcomes, so we cannot link assessment practices to clinical impact—though this relationship is well-established in the broader literature. Finally, unmeasured confounders (organizational culture, reimbursement models, available resources) may explain some observed associations.

## Conclusion

CI in HF is a substantial, underrecognized clinical challenge. This survey demonstrates that systematic assessment occurs rarely, creating a critical gap in quality of care. Barriers seem to be both behavioural and systemic. The universal educational interest and strong support for screening among active assessors indicate readiness for change. By addressing practical implementation barriers (workflow redesign, tool training, and referral pathways) tailored with targeted educational interventions, healthcare systems can close the gap. Early detection enables timely interventions that may improve adherence, reduce hospitalizations, and enhance quality of life.

## Collaborators

**Taskforce of the Hellenic Heart Failure Clinics Network:** Ioannis Alexanian, Athanasios Anadiotis, Xanthi Apostolidou, Soultana Bakaimi, Constantinos Bakogiannis, Despoina Balta, Dora Bambali, Michael Bonios, Alexandros Briasoulis, Georgios Chalikias, Eftychia Chamodraka, Christos Chasikidis, Christos Chatzieleftheriou, Argirios Dalianis, Georgios Diakakis, Leonidas Diamantopoulos, Stella Dourtsiou, Metaxia Driva, Dimitrios Farmakis, Gerasimos Filippatos, Katerina Fountoulaki, Nikolaos Fragakis, Gerasimos Gavrielatos, Maria Georgopoulou, Grigoris Giamouzis, George Giannakoulas, George Giannoglou, Nikolaos Gouliaros, Angeliki Gkouziouta, Nikolaos Kalantzis, Vasileios Kamperidis, Nikolaos Kampouridis, Iraklis Kapitsinis, Theodoros Karamitsos, Eleni Karapatsoudi, Athanasios Kartalis, Anastasia Katinioti, Konstantinos Katsilis, Anastasia Kitsiou, Anna Kotsia, Maria Kouli, Nikos Kouris, Stauroula Kosmopoulou, Emmanouil Lamprogiannakis, Stylianos Lampropoulos, Evangelos Lazaris, Ioannis Leontsinis, Athanasios Manginas, Polyxeni Mantzouratou, Spyridon Maragkoudakis, Maria Marketou, Georgios Nikitas, Petros Nikolopoulos, Paraskevi Ntoliou, Christodoulos Papadopoulos, Dimitrios Papadopoulos, Konstantinos Papadopoulos, Georgios Papanikolaou, Minas Pappas, Stefanos Papastefanou, Vasilios Sarakis, Evangelos Sdogkos, Maria Sifaki, Vasiliki Sklirou, Georgios Spyromitros, Alexia Stavrati, Ioannis Stratakis, Maria Stratinaki, Efstratios Theofilogiannakos, Maria Thodi, Konstantina Toli, Konstantinos Tsatiris, Georgios Tsinopoulos, Konstantinos Tsiptsis, Konstantinos Tsitlakidis, Dimitrios Tziakas, Elias Tsougos, Vasileios Vasilakopoulos, Vassilios Vassilikos, Athanasios Vasilopoulos, Niki Vlassopoulou, Ioannis Vogiatzis, Petros Voutas, Alexandros Xanthopoulos, Ermioni Zachariadi, Antonios Ziakas.

## Declarations

### Data Availability

No data were generated or analysed for this manuscript.

### Pre-registered Clinical Trial Number

None supplied.
